# Evolution of SILS Cholecystectomy in the Caribbean: The Direct Transfascial Puncture Technique Using Conventional Instruments without Working Ports

**DOI:** 10.1155/2014/164342

**Published:** 2014-10-02

**Authors:** Shamir O. Cawich, Dexter Thomas, Dale Hassranah, Vijay Naraynsingh

**Affiliations:** Department of Clinical Surgical Sciences, University of the West Indies, St. Augustine Campus, St. Augustine, Trinidad and Tobago

## Abstract

*Introduction.* Single incision laparoscopic cholecystectomy (SILC) has become accepted as an alternative to conventional multiport cholecystectomy. However, SILC is still limited in applicability in low resource centres due to the expense associated with specialized access platforms, curved instruments, and flexible scopes. *Presentation of Case.* We present three cases where a modified SILC technique was used with conventional instruments and no working ports. The evolution of this technique is described. *Discussion.* In order to contain cost, we used conventional instruments and three transfascial ports placed in an umbilical incision, but we noted significant instrument clashes that originated at the port platforms. Therefore, we modified our technique by omitting ports for the working instruments. The technique allowed us to exchange instruments as necessary, maximized ergonomics, and prevented collisions from the bulky port platforms. Finally, the puncture left by the instrument alone did not require fascial closure at the termination of the procedure. *Conclusion.* The direct transfascial puncture using conventional laparoscopic instruments without working ports is a feasible option that minimizes cost and increases ergonomics.

## 1. Introduction

Navarra et al. were the first to report the completion of a cholecystectomy through a single periumbilical incision in 1997 [1]. Initially, single incision laparoscopic cholecystectomy (SILC) was slow to gain traction because it was technically difficult and expensive due to need for specialized access platforms, curved instruments, and flexible tip laparoscopes. However, over the past decade SILC has become widely accepted as a feasible and safe alternative to multiport cholecystectomy. There have been increasing reports of modified SILC techniques using straight instruments [2–10] and modified access platforms [10–17].

The first SILC in the Caribbean was performed in 2009 [2]. Since this time, the technique has undergone several adaptations. We report the evolution in instrumentation and accesses in our practice of SILC in a limited-resource Caribbean setting.

## 2. Presentation of a Case

**Figure 1:**
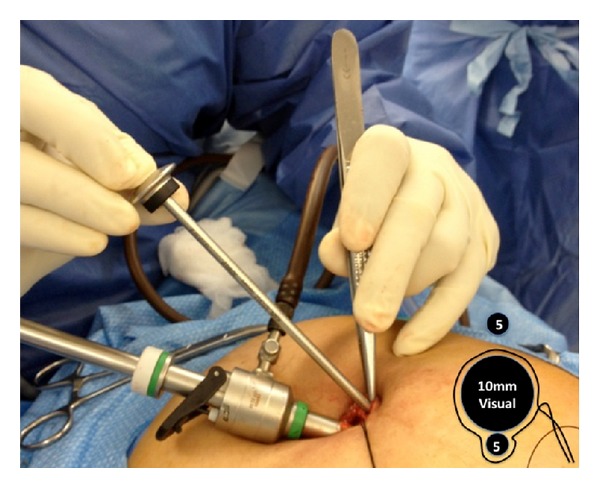
The 10 mm visual port is placed at the right side of the fascial window. A 5 mm working instrument will be passed alongside, encircled by a purse string suture (inset). A 5 mm introducer is used to puncture the fascia at the left side of the fascial window to allow a 5 mm instrument to be passed directly across the fascial tract outside the purse string suture (inset).

At our institution, three patients underwent SILC using a modified technique with conventional instruments. A 2 cm incision was created across the umbilicus. The skin was undermined in order to maximize fascial exposure. An 11 mm incision was created in the fascia at the left side of the fascial window. A purse string suture was inserted at the margins of the fascial incision using 1/0 prolene sutures. A 10 mm optical port was placed in the incision. The purse string suture was tightened to create a seal and insufflation commenced to achieve a 12 mmHg pneumoperitoneum. A 5 mm trocar introducer was then used to puncture the fascia at the right side of the wound (Figure 1). The introducer was withdrawn and a 5 mm instrument immediately advanced across the fascia through the tract. This was the most commonly utilized instrument for the surgeon and therefore would require the least “change.” In our hands, this was an electrocautery hook.

**Figure 2:**
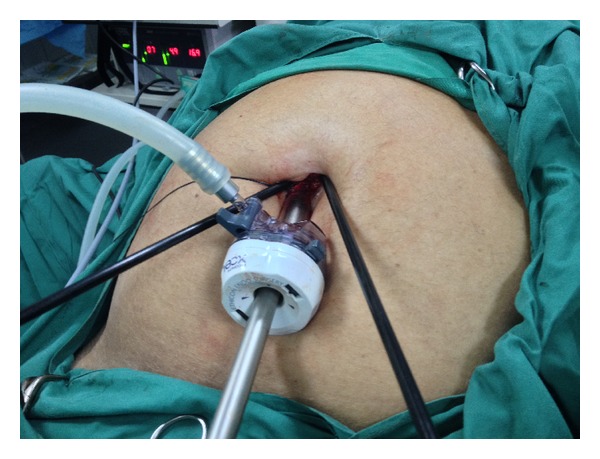
10 mm visual port is placed in the umbilical incision and a 5 mm Maryland's grasper passes beside the visual port, both encircled by a purse string suture to create a seal. A 5 mm working instrument (cautery hook) is passed directly across the fascia outside the purse string suture.

**Figure 3:**
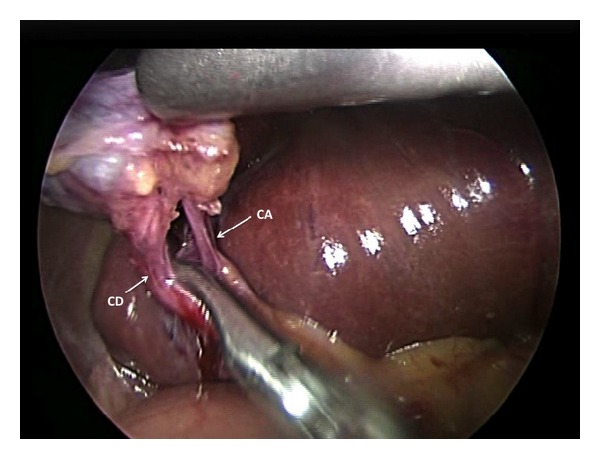
A 30° laparoscope is used to demonstrate Strasberg's critical view during the SILC. Cystic duct (CD) and cystic artery (CA) are demonstrated.

To complete the cholecystectomy, the purse string suture was relaxed and a 5 mm straight instrument was passed beside the optical port (Figure 2). The purse string was tightened, encircling both instruments to regain a seal and reestablish a pneumoperitoneum. The cholecystectomy then proceeded in a normal fashion by dissecting Calot's triangle to achieve Strasberg's critical view (Figure 3). The cystic artery and duct were then clipped using a 5 mm clip applicator passed beside the visual port within the purse string suture. After division of these structures, the cautery hook was used to dissect the gallbladder from the hepatic bed.

Three procedures were completed with a mean operating time of 42 minutes. There were no conversions to open or multiport cholecystectomies. No complications were recorded.

## 3. Discussion

The first SILC in the Caribbean was performed in Jamaica in 2009 [2]. There are several challenges preventing minimally invasive surgery from being established in this environment [18–20]. At the forefront is the fact that this is a developing nation with an underfunded health care system [18]. The public health care system cannot afford to procure access platforms, flexible scopes, or curved instruments. Therefore, our SILC technique was modified out of necessity to one using straight instruments and conventional laparoscopes [9]. Similarly, many authors report having reverted to the use of conventional instruments for SILC [2–10].

However, our technique continued to evolve since we had not found an acceptable and affordable access platform. Our first SILC was performed with donated SILS ports (Covidien Inc., Norwalk, CT, USA) but after the donated supplies depleted, we were forced to experiment with different types of accesses. To contain cost, we attempted using three reusable transfascial ports placed in an umbilical incision. The technique achieved the goal of cost-containment but it increased the technical difficulty of SILC because of instrument clashes.

We noticed that the “instrument clashes” did not involve the instruments but occurred between the port platforms. The natural progression was to omit the ports and pass the instruments directly across the fascia. Therefore, we modified the technique by omitting ports for our working instruments. By passing one instrument beside the optical port, we were able to exchange instruments during the procedure as necessary. Since this instrument was only 5 mm in diameter, a seal could be maintained by tightening the purse string suture that encircled the optical port and working instrument.

The second working instrument was passed directly across the fascia following the tract created by a puncture from a 5 mm optical trocar introducer. By omitting the working port, the bulky port platforms were not present so collisions could only originate from the instruments themselves—which were significantly smaller in diameter. This technique also allowed us to place this instrument at the right most extent of the wound, maximizing distance between the instruments. These two factors resulted in increased maneuverability of the working instruments. Although it did not fully compensate for the lack of triangulation inherent to SILC, the instruments were now in a position where we could perform intracorporeal tasks much more ergonomically. An added benefit was that the fascial defect left by the instrument alone did not require closure at the end of the procedure.

There have been many modifications of SILC accesses. Peritoneal access using multiple low profile ports which are in a single incision has been described [5, 6], but this does not completely overcome the problem of port platform collisions. Many authors have described the use of a surgical glove with conventional ports [10–14, 16, 17] or modified syringes [15] tied into the fingers. However, all descriptions of the glove ports require the use of a wound retractor ring to maintain the seal that also incurs cost. Our modification overcomes both problems since it does not require the use of working ports or wound retractors and only creates collisions from the instruments themselves.

By reducing the amount of consumables required for each operation to a single 12 mm optical port (Covidien Inc., Norwalk, CT, USA) and standard 35 cm instruments, the direct transfascial puncture technique reduces the cost of this operation. We compared this to other methods that were available for SILC using current market prices from local distributors. The cost of one SILS port (Covidien Inc., Norwalk, CT, USA) on the local market was USD $470.21. Using the glove method with one 12 mm port, two 5 mm ports, and a wound protector (Covidien Inc., Norwalk, CT, USA), the cost of the procedure amounted to USD $209.01. Using the method with a 12 mm with two 5 mm ports placed in a single umbilical incision, the cost of the procedure was USD $109.51. In comparison, using the direct transfascial puncture technique with only one 12 mm port (Covidien Inc., Norwalk, CT, USA), the cost of consumables for this procedure was only USD $37.85. Compared to all the other methods, the cost associated with the direct transfacial puncture technique resulted in cost savings.

## 4. Conclusion

The direct transfascial puncture technique using conventional laparoscopic instruments without working ports is a feasible option. It minimizes cost and increases surgeon ergonomics for SILC. However, a series with larger case volumes is needed to definitively state the efficacy of this procedure.
